# Association of neuronal injury blood marker neurofilament light chain with mild-to-moderate COVID-19

**DOI:** 10.1007/s00415-020-10050-y

**Published:** 2020-07-09

**Authors:** Markus Ameres, Susanne Brandstetter, Antoaneta A. Toncheva, Michael Kabesch, David Leppert, Jens Kuhle, Sven Wellmann

**Affiliations:** 1 University Children’s Hospital Regensburg (KUNO), Hospital St. Hedwig of the Order of St. John, University of Regensburg, Steinmetzstr. 1-3, 93049 Regensburg, Germany; 2 Research and Development Campus Regensburg (WECARE) at the Hospital St. Hedwig of the Order of St. John, University of Regensburg, Regensburg, Germany; 3 Neurologic Clinic and Policlinic, Departments of Medicine, Biomedicine and Clinical Research, University Hospital Basel, University of Basel, Basel, Switzerland

Dear Sir,

Even though the coronavirus disease 2019 (COVID-19) affects primarily the respiratory system some reports describe nervous system involvement as well [1–3]. Headache and anosmia have been frequently described as neurological symptoms of mild-to-moderate COVID-19 but a direct impact of COVID-19 on neuronal integrity has not been clarified yet [4]. Therefore, a neuronal biomarker would be extremely useful to elucidate neuro-axonal injury during an infection with Severe Acute Respiratory Syndrome Coronavirus-2 (SARS-CoV-2) and in the post-infection follow-up period. Serum neurofilament light chain (sNfL) has recently been considered as a specific biomarker to quantitate neuro-axonal damage in several disorders of the peripheral and central nervous system [5]. Hence, sNfL might also serve as a sensitive screening and follow-up marker for neuronal injury in COVID-19 patients.

We conducted a prospective cohort study in 100 healthcare workers (84 females, 16 males) following a COVID-19 outbreak in a major German children's and women's hospital [6]. The Ethics Committee of the University of Regensburg approved the study (file-number: 20-1767-101), and written informed consent was obtained from all study participants. They were categorized according to their SARS-CoV-2 infection status, *n* = 28 tested positive, *n* = 72 negative in PCR-based viral RNA amplification from nasopharyngeal swabs (Xpert© Xpress SARS-CoV-2, Cepheid) [5]. To preserve anonymity of study participants, age was assessed in three categories (18–35 years *n* = 33, 36–50 years *n* = 37 and 51–65 years *n* = 30) [7]. sNfL concentrations were measured using the single molecule array (Simoa) NF-light^®^ kit on the HD-X Analyzer (Quanterix, Lexington, MA) [5]. First, descriptive statistics were calculated. Then, a multivariable linear regression model was fitted with sNfL as dependent variable and with sex, age and COVID-19 status as independent variables.

All COVID-19 patients had mild-to-moderate symptoms and recovered after 1–3 weeks and showed no or only minor neurological symptoms, including anosmia and headache. First, sNfL measurement was done in COVID-19 patients 23 days (median, IQR 21–26) after onset of disease. sNfL levels for COVID-19 patients and for controls, stratified for age group, are depicted in Fig. 1. Median and interquartile range for COVID-19 patients were 4.5 pg/ml [IQR 3.7–5.7] for the age group 18–35 years, 9.6 [6.5–11.3] for the age group 36–50 years, and 11.6 [8.4–18.3] for the age group 51–65 years, respectively. sNFL levels for controls were 4.4 [3.5–5.5] for the youngest group, 6.8 [5.6–8.8] for the group 36–50 years, and 9.6 [8.2–11.2] for the oldest group (Table 1).

**Fig. 1:**
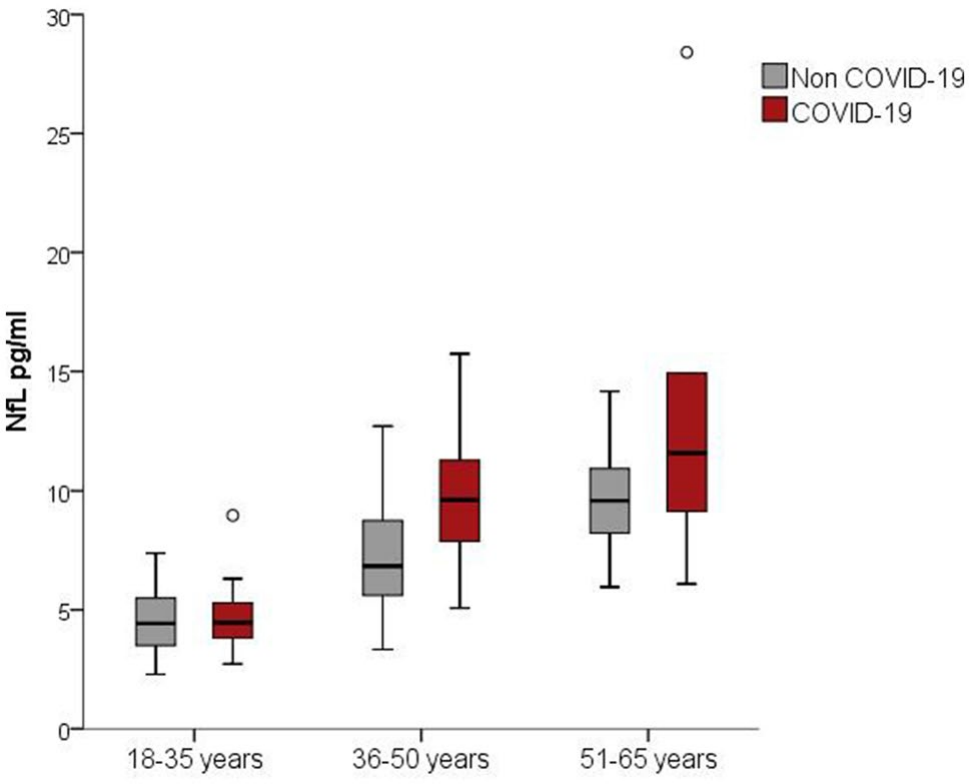
Boxplots of sNfL concentrations in COVID-19 and control cases stratified by age group. Of note, COVID-19 status (*p* = 0.005) and age group (*p* < 0.001) are significantly associated with sNfL values in a multivariable linear regression analysis of sex, age and COVID-19 status

**Table 1:** Baseline characteristics of study participants stratified by COVID-19 status

	Non COVID-19 (*n* = 72)	COVID-19 (*n* = 28)
Female (*N*, %)	59 (81.9)	25 (89.3)
Male (*N*, %)	13 (18.1)	3 (10.7)
Age group 18–35 years (*N*, %)	20 (27.7)	13 (46.4)
Age group 36–50 years (*N*, %)	28 (38.9)	9 (32.14)
Age group 51–65 years (*N*, %)	24 (33.3)	6 (21.4)
Respiratory symptoms (*N*, %)		17 (60.7)
Neurological symptoms (*N*, %)		21 (75.0)

Notes: respiratory symptoms included cough and shortness of breath; neurological symptoms included headache and anosmia

Since sNFL levels are highly dependent on age [8] the association between COVID-19 status and sNFL was determined using a multivariable linear regression model with COVID-19 status, age and sex as independent variables. This analysis revealed that COVID-19 status was significantly associated with sNfL (*b* = 1.87; *p* = 0.005) when controlling for age and sex (Table 2). In COVID-19 patients with two sNfL measurements (*n* = 16, time span between the measurements was median 35 days, range 29–36 days), sNfL levels were highly correlated (*r* = 0.96).

**Table 2:** Multivariable linear regression analysis of sex, age and COVID-19 status on sNfL

	*b*	SE B	*β*	*p*
Sex (female)	− 0.02	0.84	− 0.00	0.981
Age group 18–35 years	Reference category			
Age group 36–50 years	3.38	0.72	0.44	< 0.001
Age group 51–65 years	6.10	0.74	0.76	< 0.001
COVID-19	1.87	0.65	0.23	0.005

Notes: *n* = 100. Nagelkerke’s *R*^2^ = 0.45; *b* regression coefficient, *SE B* standard error (regression coefficient), *β* standardized regression coefficient, *p* significance value

NfL is a highly specific structural protein of neurons and elevated levels of sNfL are recognized as measures of acute or chronic neuro-axonal damage [5]. Our results from a study in health care workers without known co-morbidities indicate that mild-to-moderate COVID-19 is associated with increased sNfL levels. Neurologic symptoms and complications in patients with SARS-CoV-2 infection have been reported by the first available studies during SARS-CoV-2 pandemic [1, 2]. However, these studies are restricted to hospitalized patients and, therefore, represent a population more likely to have severe neurological manifestations for a variety of reasons. Our results indicate for the first time that COVID-19 may affect the neuro-axonal integrity also in adults with a mild-to-moderate course of the disease. This new evidence for a more general neuro-destructive capability of SARS-CoV-2 also in mild-to-moderate COVID-19 patients should raise awareness for potential long-term neurologic sequelae following COVID-19. Of note, our study includes only a limited number of patients. In addition, information on participants’ age was collected using very broad categories and we cannot exclude that there were age differences between COVID-19 patients and controls not accounted for in the statistical adjustment using age groups. To draw further conclusions, additional studies on sNfL and COVID-19 are needed.

## Data Availability

On request.
